# Paraspinal compartment syndrome after minimally invasive thoracolumbar fixation: a case report

**DOI:** 10.1186/s13256-025-05708-y

**Published:** 2025-11-27

**Authors:** Hongxing Zhang, Junjie Du, Tengfei Li, Zhihuang Zheng, Han Wang, Zhiqiang Chen, Tianqi Li, Bowen Xie, Yufei Chen, Deshui Yu, Fengyuan Yang, Ye Peng

**Affiliations:** 1 Department of Orthopedics, Air Force Medical Center of PLA, Beijing, 100142 China; 2 Air Force Clinical College, The Fifth School of Clinical Medicine, Anhui Medical University, Anhui, 230032 China; 3 Graduate School of Medicine, China Medical University, Shenyang, 110122 China; 4 Department of Orthopedics and Traumatology, The First Teaching Hospital of Tianjin University of Traditional Chinese Medicine, Tianjin, 300193 China; 5 Beijing Jishuitan Hospital, Capital Medical University, Beijing, 100035 China; 6 Division of Surgery and Interventional Science, University College London, London, NW3 2PF UK

**Keywords:** Paraspinal compartment syndrome, Minimally invasive spine surgery, Percutaneous pedicle screw fixation, Thoracolumbar compression fracture, Postoperative wound complications, Surgical debridement, Avascular muscle necrosis, Case report

## Abstract

**Background:**

Minimally invasive spinal surgery has revolutionized the treatment of traumatic thoracolumbar compression fractures. However, rare and potentially serious complications, such as paraspinal compartment syndrome, may still occur.

**Case presentation:**

A 20-year-old Chinese male soldier sustained a T12 compression fracture and underwent successful minimally invasive percutaneous pedicle screw fixation from T11 to L1. The initial postoperative course was uneventful. On postoperative day 16, he developed severe back pain, serous fluid discharge, and localized paraspinal swelling. This prompted readmission and emergency surgical debridement. Intraoperative findings revealed pale, avascular, and noncontractile paravertebral muscles bilaterally. Pathological examination confirmed skeletal muscle necrosis, establishing the diagnosis of thoracolumbar paravertebral myofascial compartment syndrome. Aggressive management with serial debridements, negative-pressure wound therapy, and appropriate antibiotic therapy led to complete wound healing and full recovery, allowing him to resume military training.

**Conclusion:**

This case underscores the critical importance of early recognition and prompt surgical intervention in managing paraspinal compartment syndrome. Even in minimally invasive spinal surgery, enhanced intraoperative measures and vigilant postoperative monitoring are necessary to prevent such rare but potentially devastating complications.

## Background

Minimally invasive percutaneous pedicle screw fixation (MIS-PSF) has become a widely adopted method for stabilizing thoracolumbar compression fractures, offering advantages such as reduced soft tissue injury, decreased intraoperative blood loss, and faster recovery compared with traditional open surgery [1].

Despite these benefits, rare but serious complications can occur, including paraspinal compartment syndrome (PCS). PCS arises when increased pressure within the fascial compartments of the lumbar paravertebral muscles leads to ischemia and potential muscle necrosis [2]. While compartment syndromes are well-documented in extremities, PCS is exceedingly uncommon [3]. A recent review identified only 22 reported cases of PCS in the English-language literature, with the majority being isolated case reports or small case series [2]. Notably, PCS has rarely been described as a complication of spine surgery, and reports following minimally invasive spinal fixation are especially scarce [2, 3].

In this case report, we describe a young, healthy patient who developed acute PCS after MIS-PSF for a thoracolumbar fracture. We analyze the underlying pathophysiology, risk factors, and critical steps in diagnosis and management. We aim to highlight that, although PCS is rare, its potential for severe muscle and nerve injury necessitates early recognition and prompt treatment, even in the context of minimally invasive spine surgery.

## Case presentation

### Case background and initial consultation

**Fig. 1:**
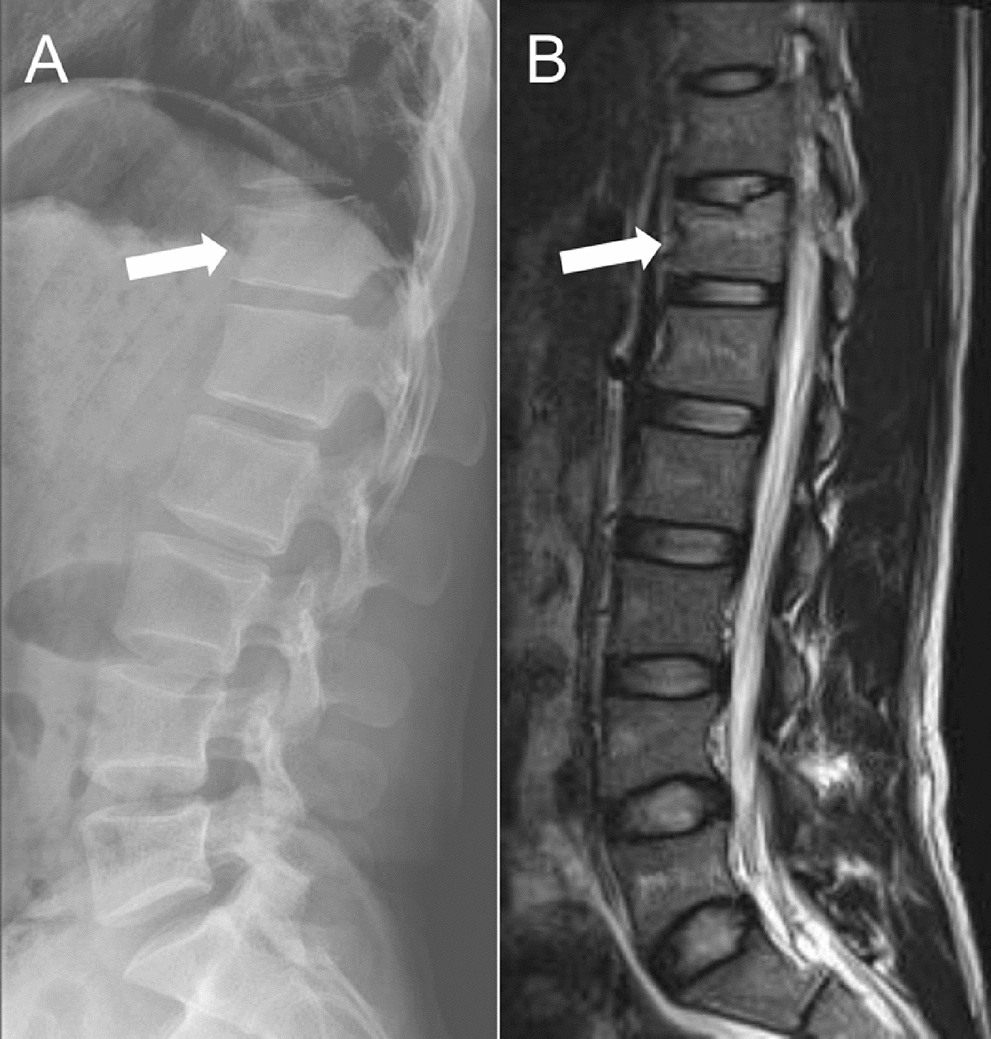
Preoperative imaging assessment of the T12 vertebral compression fracture. **A** An anteroposterior radiograph of the thoracolumbar spine reveals an anterior wedge compression fracture at T12 (arrow), without posterior bone displacement or compromise of the spinal canal. **B** Sagittal T2-weighted MRI of the same region confirms an acute T12 fracture, demonstrating bone marrow edema at the fracture site (arrow), with an intact posterior ligamentous complex and no evidence of epidural hematoma or neural compression.

A 20-year-old Chinese male soldier with no significant medical history sustained a fall during military training, landing directly on his hip. He experienced immediate severe back pain and had difficulty moving. A primary neurological examination in the field revealed no evidence of sciatica or other neurologic deficits. The patient was then referred to our hospital for further evaluation. Imaging on admission confirmed a compression fracture of the T12 vertebra (non-osteoporotic) with no spinal cord compression (X-ray, Fig. 1A; magnetic resonance imaging (MRI), Fig. 1B). Because he was young and the fracture was stable and non-osteoporotic, cement augmentation (vertebroplasty/kyphoplasty) was not indicated. He also declined conservative management (which would have required approximately 6 weeks of bed rest) owing to his military duties. Therefore, after discussing the available options, the patient opted for surgical intervention as the definitive treatment.

### Initial surgery and early recovery

Three days after the injury, the patient underwent minimally invasive percutaneous pedicle screw fixation (MIS-PSF) spanning T11, T12, and L1 under general anesthesia. The surgery was performed through two small bilateral paraspinal incisions, allowing percutaneous screw placement without a large midline incision. The procedure was completed in 100 min with an estimated blood loss of only 40 mL. No surgical drain was placed, as there was no significant intraoperative bleeding and only minimal postoperative fluid was anticipated. By postoperative day 2, the patient was able to ambulate with a waist support. He was discharged from the hospital on postoperative day 7, neurologically intact, with the incision sites healing well and showing no signs of infection or other complications.

### Early postoperative complication and readmission

On postoperative day 16, the patient returned to our hospital with acute severe back pain. He had developed an oozing serous fluid discharge from the right paraspinal incision site, accompanied by localized swelling and mild tenderness in the surrounding area. These findings prompted immediate readmission for evaluation of a wound complication. On examination, his body temperature was 36.8 °C (afebrile). Notably, he still had no motor or reflex deficits. However, there was firm swelling (palpable tension) in the paraspinal muscles, and sensation was decreased (hypoesthesia) over the T11–L1 dermatomes. During his postoperative course up to that point, the patient had adhered to medical advice by resting and avoiding strenuous activity, which likely contributed to the absence of more severe systemic symptoms. Laboratory investigations at readmission showed a normal white blood cell (WBC) count and procalcitonin (PCT) level, while the erythrocyte sedimentation rate (ESR), C-reactive protein (CRP), and creatine kinase (CK) were mildly elevated (see Table 1 for serial values). These clinical and laboratory features were initially interpreted as most consistent with a poorly healed surgical wound with a suspected incisional infection. However, the absence of fever or significant leukocytosis—combined with the presence of tense swelling and hypoesthesia—also raised the possibility that a deeper process, such as an evolving paraspinal compartment syndrome, could be developing in addition to a superficial wound issue.

**Table 1:** Serial trends in the patient’s key laboratory parameters during the perioperative course

Days since readmission	Laboratory test results					
	WBC (× 10^9^/L)	ESR (mm/h)	CRP (mg/L)	PCT (ng/ml)	CK (U/L)	Bacterial culture
1 day	6.0	36↑	51.00↑	0.024	412↑	^–^
7 days	6.0	14	7.92	0.020	351↑	Negative*
10 days	5.3	13	7.44	0.020	257	Negative*
14 days	5.2	10	2.65	0.023	131	–
21 days	5.2	10	< 1.00	0.020	75	–
35 days	5.7	8	4.00	0.020	52	–

*WBC* white blood cell;* ESR* erythrocyte sedimentation rate;* CRP* C-reactive protein; *CK* creatine kinase;* PCT* procalcitonin. Reference ranges (institutional): WBC 3.5–9.5 × 10⁹/L; ESR 0–15 mm/h; CRP 0–10 mg/L; CK 50–310 U/L; PCT < 0.05 μg/L. Values exceeding the normal range are marked with an upward arrow (↑). An asterisk (*) denotes a time point at which a wound fluid culture was obtained (on those days, bacterial cultures showed no growth)

### First debridement and wound management

An urgent wound debridement and exploration was performed on the day of readmission, under local anesthesia with mild sedation. The right-side incision was extended to allow adequate exposure, and upon opening the incision, a collection of serous fluid and areas of necrotic tissue were found in the underlying paravertebral muscle and fascia. All necrotic tissue in the right paraspinal region was meticulously excised until healthy, bleeding muscle was visible. The left incision site appeared normal at this time and was not opened during the first debridement. Thorough hemostasis was achieved, followed by copious irrigation of the wound with hydrogen peroxide and a 2-min soak with povidone–iodine solution (iodophor) to disinfect the cavity. After completing the irrigation, a single closed-suction drainage tube was placed through the right incision. The pedicle screw instrumentation was left in place because there was no evidence of deep infection involving the hardware, no fracture nonunion, and the spine remained stable. The muscle and subcutaneous layers were loosely approximated, and the skin was intermittently sutured around the drain, with a sterile dressing applied. No tissue or fluid samples were collected for bacterial culture during this first debridement, as the surgical team initially believed it to be a superficial wound issue. Postoperatively, the patient was started on broad-spectrum intravenous antibiotics (cefuroxime 750 mg three times daily) to cover potential infection. Meticulous wound care was performed with dressing changes three times daily using antiseptic technique (povidone–iodine disinfectant and normal saline irrigation). The closed-suction drain yielded approximately 20 mL of fluid per day in the first few days, and the output gradually decreased over the next week.

### Persistent wound issues and second debridement

**Fig. 2:**
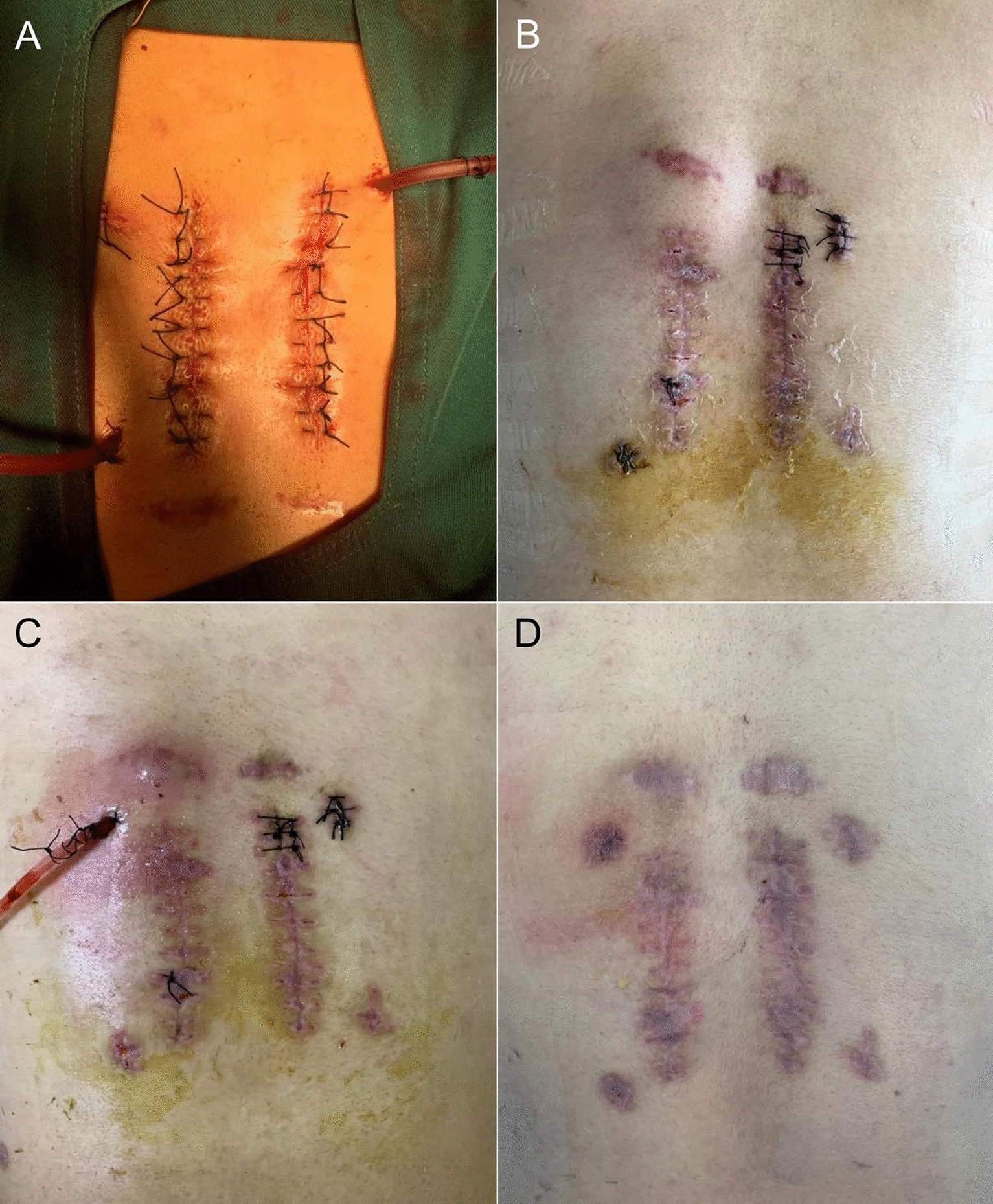
Postoperative wound management and healing progression following the second-stage surgical debridement. **A** Intraoperative appearance at the second debridement: the bilateral paraspinal incisions were extended, and two closed-suction drains were placed to evacuate the extensive subfascial fluid collections. **B** One week after removal of the left-side drain, the left incision site showed markedly reduced erythema and subcutaneous edema, with only minimal serous exudate. **C** Introduction of a negative-pressure wound therapy dressing and suction tube on the left side provided continuous drainage, preventing re-accumulation of fluid. **D** By 2 weeks after suture removal, both incisions had fully epithelialized with no signs of wound dehiscence, drainage, or infection

After 1 week of this treatment (postoperative day 23), the wound on the left side (opposite the original drainage site) began to show subtle swelling and signs of compromised healing. Gentle pressure over the left paraspinal incision caused yellowish fluid to ooze out, suggesting a new pocket of fluid or ongoing tissue breakdown on that side. This finding prompted a second surgical debridement and exploration under local anesthesia with conscious sedation, as the patient had diminished pain sensation in the affected area owing to the evolving compartment syndrome. In this second procedure, both the left and right paraspinal incisions were opened fully and extended as needed to access the underlying compartments. Strikingly, the bilateral paravertebral muscles were found to be pale, avascular, and noncontractile—findings consistent with skeletal muscle necrosis due to compartment syndrome. Extensive necrotic muscle tissue was identified in both paraspinal regions and was surgically removed (bilateral paraspinal fasciotomy and debridement) until only viable muscle with active bleeding remained. Multiple samples of the necrotic muscle were collected and sent for pathological analysis, and a 10-mL sample of wound fluid was sent for bacterial culture (the first culture obtained in this case). On inspection, the pedicle screw hardware was solidly fixed, and the spine remained stable, confirming that there was no mechanical issue with the instrumentation. The wound beds on both sides were thoroughly irrigated with hydrogen peroxide and povidone–iodine antiseptic (similar to the first debridement). One closed-suction drain was placed in each paraspinal incision tract, and the skin on both sides was loosely resutured, as shown in Fig. 2A.

### Definitive diagnosis and further management

**Fig. 3:**
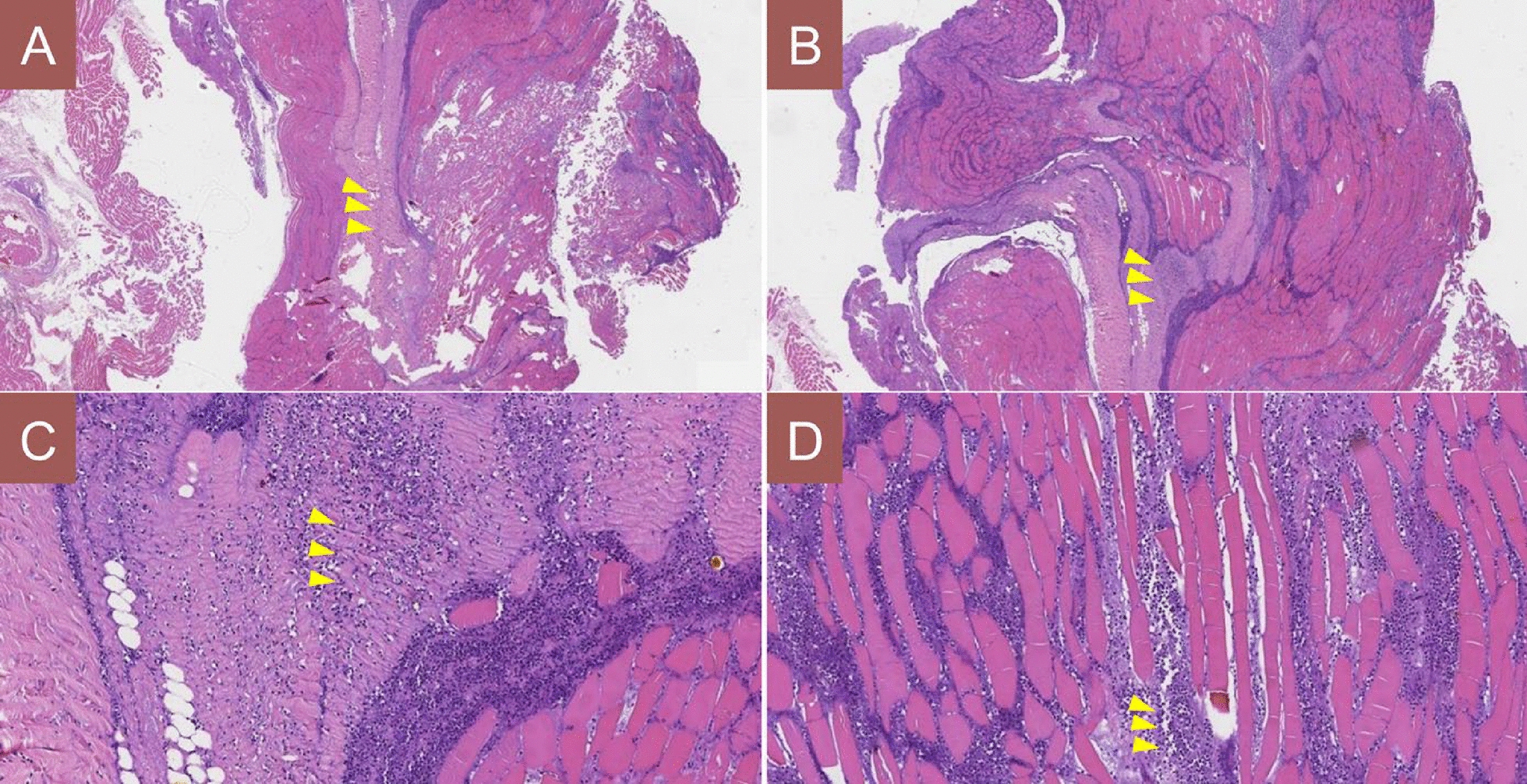
Histopathological confirmation of paraspinal skeletal muscle necrosis with acute inflammation. **A**, **B** Low-power hematoxylin and eosin staining at 10× magnification demonstrates extensive coagulative necrosis of skeletal muscle fibers (arrows), evident by the loss of normal cross-striations and homogeneous eosinophilic appearance of the fiber cytoplasm. Adjacent areas of viable muscle show interstitial edema and an infiltration of neutrophils. **C**, **D** Higher-power views (hematoxylin and eosin, 20×) highlight degenerative changes in the muscle cells, including nuclear pyknosis and focal karyorrhexis, accompanied by dense aggregates of neutrophilic inflammatory exudate (arrows)

**Fig. 4:**
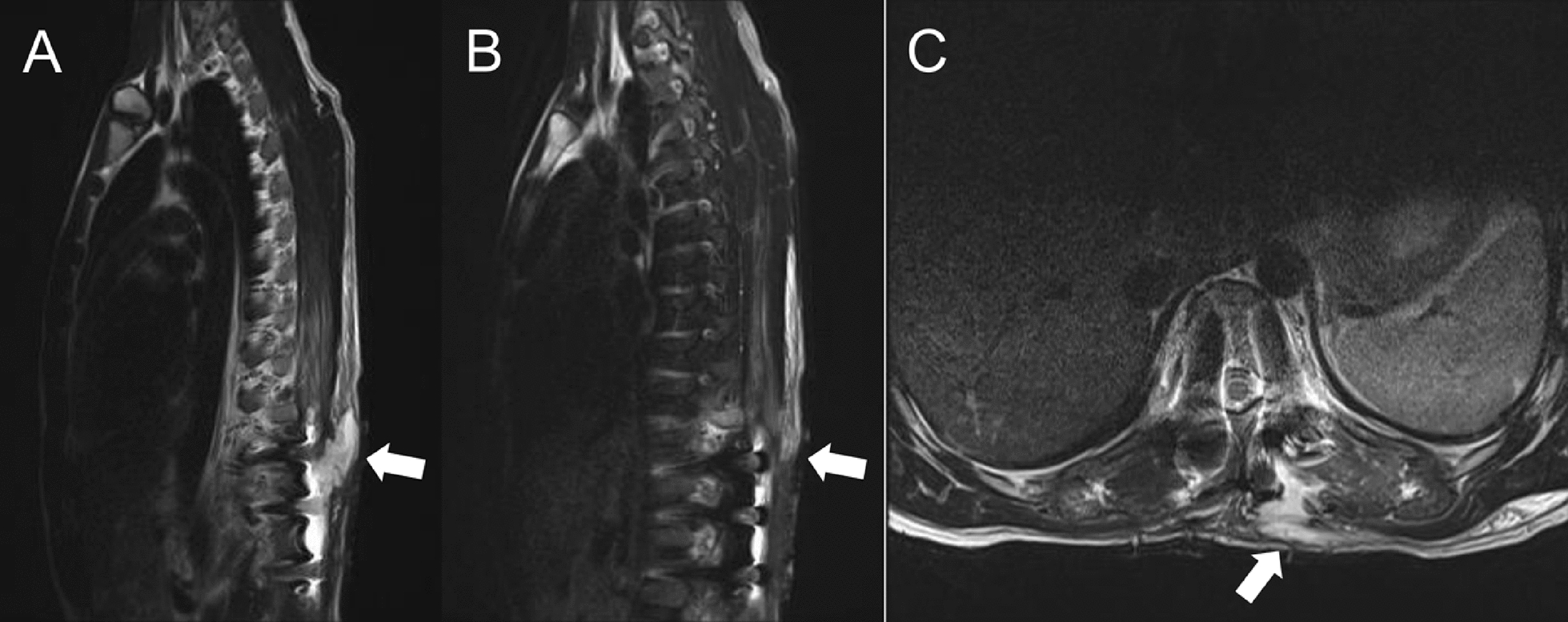
Magnetic resonance imaging visualization of postoperative fluid accumulation in the paraspinal soft tissues. **A**, **B** Sagittal fat-saturated T2-weighted magnetic resonance imaging sequences of the thoracolumbar spine demonstrate an asymmetric hyperintense fluid collection on the left side (**A**, arrow) involving subcutaneous and intramuscular compartments, compared with the appearance of the contralateral (right) side (**B**). **C** An axial T2-weighted magnetic resonance image through the T12 level localizes the fluid collection (arrow) to the left paravertebral musculature. These imaging characteristics are consistent with a postoperative seroma (sterile fluid accumulation) or reactive edema in the soft tissues

In view of the extensive bilateral muscle necrosis and the need for prolonged open wound care, the antibiotic regimen was escalated postoperatively to include intravenous vancomycin (to ensure coverage of any possible deep infection), even though there was still no definitive evidence of bacterial involvement. Follow-up laboratory tests after the second debridement showed that the WBC count, ESR, CRP, PCT, and CK had all returned to normal levels, and cultures of the wound drainage fluid showed no bacterial growth (Table 1). Specifically, the initial culture obtained during the second debridement (postoperative day 23) showed no organism growth, and a repeat culture of fluid aspirated 3 days later (postoperative day 26) was also negative. The pathological examination of the resected muscle tissue confirmed acute skeletal muscle necrosis with inflammatory exudates (Fig. 3), but no organisms were identified, supporting a diagnosis of sterile (ischemic) necrosis. On the basis of these findings, the patient’s condition was definitively diagnosed as a thoracolumbar paravertebral myofascial compartment syndrome, leading to ischemic necrosis of the paraspinal muscles. The bilateral drainage tubes were left in place until the output from each side had decreased to less than 5 mL per day; they were then gradually removed. About 1 week after removal of the left-side drain, however, the patient noted a recurrent painful swelling at the superior aspect of the left incision with a slight fluid discharge (Fig. 2B). Ultrasound examination of the area revealed a localized fluid collection under the left incision site, and a thoracic spine MRI demonstrated significant fluid accumulation within the left paraspinal muscle layers and subcutaneous tissue adjacent to the instrumentation (Fig. 4). To address this persistent fluid collection, a vacuum-assisted negative-pressure wound therapy (NPWT) system was applied under local anesthesia through a new small opening near the left incision (Fig. 2C). The aggressive wound care protocol continued with thrice-daily dressing changes using povidone–iodine and saline irrigation.

### Final outcome and follow-up

**Fig. 5:**
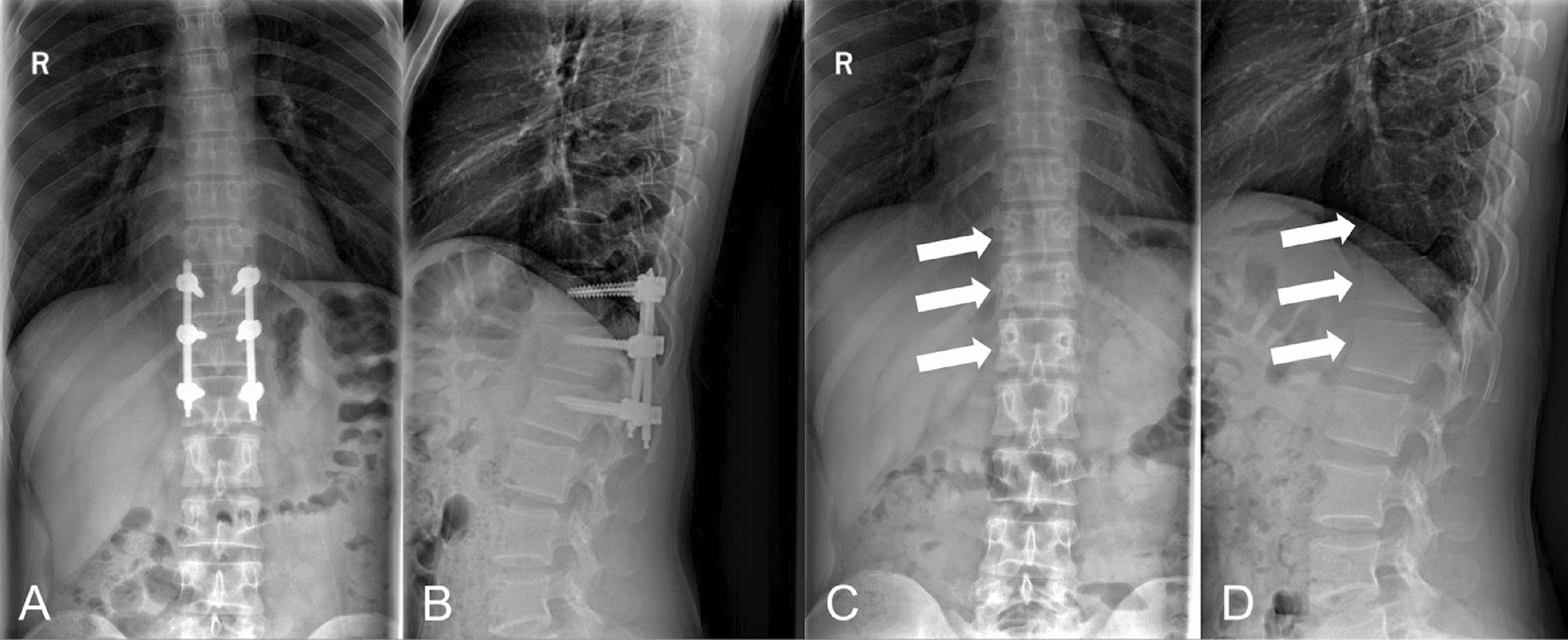
Radiographic follow-up illustrating spinal implant integrity and the results after hardware removal. **A**, **B** Anteroposterior and lateral radiographs obtained at approximately 1.5 years after the initial fixation show intact pedicle screw and rod hardware with solid bony fusion across the T12 fracture site, confirming successful stabilization. **C**, **D** Anteroposterior and lateral radiographs after elective removal of the instrumentation (performed at 1.5 years post-injury) demonstrate maintained spinal alignment and clear evidence of trabecular bone remodeling filling in the former screw tracts (arrows). No residual lucency, hardware failure, or spinal instability is observed following implant removal

**Fig. 6:**
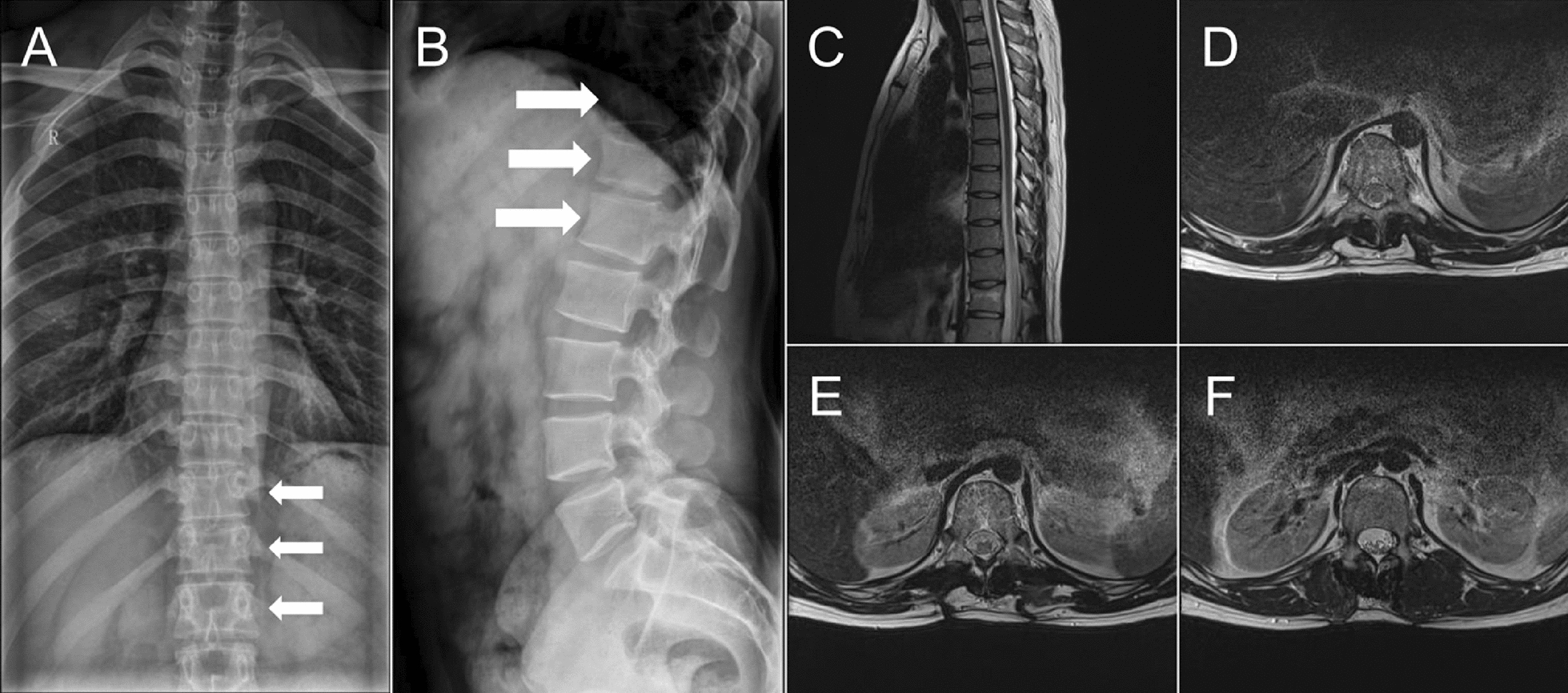
Long-term imaging at 2.5 years post-injury demonstrates complete osseous integration and adaptive muscle recovery. **A**, **B** Axial and sagittal radiograph images show that the previous pedicle screw channels have fully ossified, with no remaining radiolucent tracks (arrows). **C** A sagittal T2-weighted magnetic resonance imaging slice reveals robust hypertrophy of the paraspinal musculature surrounding the once-compromised T12 level. **D**–**F** Serial axial T2-weighted magnetic resonance imaging sections through the T12 area confirm circumferential regeneration and thickening of the paraspinal muscles encircling the stabilized segment, correlating with the patient’s functional recovery

The negative-pressure drain was maintained for 1 week and then removed once its output had become minimal. Two weeks later, all remaining skin sutures were removed, and by that time the wounds on both sides had fully healed (Fig. 2D). After approximately 4 weeks of inpatient treatment for the postoperative wound complications, the patient was discharged home. He was able to resume normal daily activities without discomfort and was closely followed on an outpatient basis. At a follow-up visit 1.5 years after the initial fixation surgery, the patient elected to have the pedicle screw instrumentation removed in hopes of reducing any risk of future hardware-related problems and restoring some natural spinal mobility (Fig. 5). The hardware removal procedure was uneventful. Following removal of the hardware, the patient recovered well and returned to full military training activities. At the final follow-up, 2.5 years after the initial injury (imaging shown in Fig. 6), he remained in excellent physical condition with no neurological deficits or functional limitations. His thoracolumbar spine was pain-free, stable, and properly aligned on imaging, and the patient was fully fit for all required military training.

## Discussion

This case of acute paraspinal compartment syndrome (PCS) after minimally invasive posterior spine fixation highlights that PCS, though rare, can be devastating even in “low-risk” procedures. In contrast to the well-known chronic exertional form (type II PCS in athletes), acute PCS can arise swiftly after trauma, intense exertion, or surgery [4]. Although minimally invasive spine techniques generally minimize muscle trauma [5], they still involve retractor-mediated compression and forceful instrumentation in a tight fascial space. PCS developed following minimally invasive percutaneous pedicle screw fixation (MIS-PSF) for a traumatic T12 fracture, highlighting the need for heightened vigilance. Any unexpected severe back pain or neurological change after surgery should prompt consideration of PCS.

### Pathogenesis of PCS in minimally invasive surgery (MIS)

Anatomic factors make the thoracolumbar paraspinal muscles prone to compartment syndrome [6]. The deep paraspinal muscles lie within rigid osseous and fascial boundaries, so even modest swelling can dramatically raise intramuscular pressure. Minimally invasive approaches utilize tubular retractors and sequential dilators, which concentrate stress on the muscle fibers without widely stripping them from their attachments. Prolonged retraction or forceful screw placement can transiently obstruct local blood flow. Sudhir *et al.* [5] reported that percutaneous pedicle screw insertion likely damaged dorsal intercostal vessels supplying the longissimus muscle, causing acute hemorrhage and edema within the thoracolumbar fascial sleeve and resultant muscle ischemia. Likewise, injury to posterior rami vessels or nerves during surgery could diminish collateral perfusion to the erector spinae. These mechanical and neural insults, combined with the unyielding circumferential compartment around the paraspinal muscles, predispose to ischemia–reperfusion injury. In a closed compartment, such as the paraspinal space, even the absence of a postoperative drain (allowing fluid to accumulate) or premature vigorous activity can abruptly increase intramuscular pressure. Prior studies document that resting paraspinal pressures are normally very low (approximately 3 to 8 mmHg) but can rise dramatically when PCS develops—values exceeding 70 mmHg (and even up to approximately 150 mmHg in extreme cases) have been reported [6]. In summary, MIS-related PCS likely results from a multifactorial interplay of localized ischemia (from prolonged compression and traction), denervation, and vascular compromise within the tightly bound paraspinal compartment [5, 6].

### Diagnostic evaluation and challenges

The patient’s presentation—sudden, severe back pain with board-like paraspinal rigidity and dermatomal hypoesthesia (T11–L1) after surgery—was highly concerning for an ischemic compartment etiology. Importantly, systemic inflammatory markers remained normal and wound cultures were negative, while serum creatine kinase (CK) was elevated above normal limits (peaking at 412 U/L on the day of readmission, versus a normal upper limit of 310 U/L). This pattern of excruciating focal pain with even a mild CK rise (in the absence of fever or infection) is strongly suggestive of muscle ischemia consistent with paraspinal compartment syndrome. Notably, acute PCS typically features far more dramatic CK elevations (often > 5000 U/L) [6], and a literature review of 17 cases reported peak CK values ranging from approximately 5000 up to 350,000 U/L [7]. Our patient’s comparatively modest CK increase still indicated significant muscle injury, albeit less extreme, which may reflect the benefit of early intervention or the involvement of a smaller muscle volume.

This clinical picture aligns with classic descriptions by Nathan *et al.* [4], who characterize acute PCS as excruciating, localized back pain accompanied by board-like rigidity of the paraspinal muscles and localized sensory loss, typically arising within hours of the inciting event. In our case, however, the compartment syndrome became evident only about 16 days post-injury and surgery—a markedly delayed presentation compared with the usual onset within the first day or two. This subacute timing underscores that vigilance must be maintained even well beyond the immediate postoperative period. Advanced imaging can support the diagnosis: MRI (or ultrasound) of the spine will show diffuse edema and fluid collection within the affected muscle compartment (hyperintense T2 signal), which helps distinguish PCS from other causes of postoperative back pain [6]. In our patient, MRI demonstrated extensive bilateral paravertebral muscle edema and fluid, corroborating the clinical suspicion and highlighting that a compartment syndrome (rather than simple postoperative musculoskeletal pain or typical swelling) was present.

Because the paraspinal muscle compartments are deep and not easily accessible like limb compartments, direct compartment pressure measurement—the gold standard for diagnosing limb compartment syndrome—is often impractical in acute PCS. Nathan *et al.* [4] note that a thorough clinical examination combined with imaging findings can suffice for diagnosis, reserving invasive pressure monitoring for only the most unclear cases. In our patient, a definitive diagnosis was obtained intraoperatively: the paraspinal muscles appeared pale and noncontractile (consistent with acute ischemia), and subsequent histopathology confirmed necrotic muscle tissue. In hindsight, obtaining a tissue culture during the first debridement might have been informative; however, at that time, the clinical picture was thought to represent a superficial wound issue, and empiric antibiotics were started. This underscores how PCS can masquerade as an infection or routine postoperative inflammation. This case thus illustrates the diagnostic complexity of PCS, which can easily be mistaken for more common postoperative issues. It underscores the need to integrate multiple clues—disproportionate pain unrelieved by analgesics, persistent paraspinal rigidity or focal hypoesthesia, an isolated CK elevation with normal inflammatory markers, and imaging evidence of muscle edema—all of which point toward acute PCS rather than typical post-surgical pain or infection.

In summary, a high index of suspicion is required in any high-risk setting, as timely recognition of PCS is critical. MRI and clinical examination are the mainstays of diagnosis. If acute PCS is strongly suspected on the basis of clinical and imaging findings, one should proceed directly to urgent surgical decompression (fasciotomy) without waiting for compartment pressure measurements [4, 5]. This proactive approach is crucial for preventing irreversible muscle ischemia and improving patient outcomes.

### Management strategies and outcomes

Early surgical decompression is the cornerstone of treatment for acute PCS. Analogous to extremity compartment syndromes (in which delays beyond 24–48 h can cause irreversible myonecrosis), prompt paraspinal fasciotomy can salvage muscle tissue and prevent permanent neurologic deficits [4, 5]. In this case, initial conservative measures (aggressive pain control, antibiotics, and wound care) were reasonable to rule out a superficial infection, but once underlying muscle necrosis was recognized, nonoperative management was insufficient. Accordingly, bilateral paraspinal fasciotomies through extensile incisions were performed, allowing decompression and thorough debridement of all necrotic muscle. Negative-pressure wound therapy (using a vacuum-assisted closure dressing) was then utilized to manage edema, promote granulation tissue formation, and prepare the wound beds for eventual closure. This aggressive approach mirrored the “time is muscle” principle: as Sudhir *et al.* emphasize, urgent decompression even after seemingly small-scale spine procedures (such as percutaneous pedicle screw fixation) can prevent the progression to avascular necrosis of muscle [5]. In our patient, this strategy yielded an excellent functional recovery—he returned to normal activities within a month and was fully recovered by 2.5 years postoperatively. Notably, published PCS cases that were managed nonoperatively tend to have much more protracted recoveries (on the order of many weeks to over a year) and often suffer chronic pain or muscle fibrosis [4, 5]. Therefore, an acute PCS with any evidence of muscle compromise or necrosis warrants expedited fasciotomy and serial debridements, whereas a milder “incipient” presentation (with no frank necrosis yet) might initially be observed with very close monitoring of symptoms, laboratory values, and imaging.

### Proposed diagnostic and management algorithm

To assist clinicians, we propose the following approach for patients after MIS spine surgery who may be at risk for PCS:

- Maintain a high index of suspicion: Be alert for disproportionate back pain, abnormally tense (“wooden”) paraspinal muscles, new-onset sensory changes over paraspinal dermatomes, or even unexpected sterile, serous wound drainage. Any of these red flags should prompt immediate evaluation [4, 5].
- Obtain urgent imaging and labs: Perform an MRI of the thoracolumbar spine to look for diffuse paraspinal muscle edema or fluid collection within the compartment [4]. Simultaneously, check the patient’s CK level (expect a marked rise in PCS) and assess renal function to monitor for rhabdomyolysis-induced kidney injury.
- Interpret findings comprehensively: If the clinical exam and MRI strongly indicate compartment syndrome (for example, bilateral paraspinal T2 hyperintensity on imaging coupled with focal hypoesthesia), proceed toward fasciotomy. If the picture is equivocal, consider measuring the intramuscular compartment pressure. For example, one can use a Stryker needle device to directly measure pressure and calculate the ΔP (delta pressure = diastolic blood pressure minus compartment pressure); a Δ*P* < 30 mmHg is generally diagnostic of compartment syndrome [6].
- Proceed to fasciotomy when indicated: Any confirmed or highly suspected PCS should be treated with prompt surgical decompression. Make generous bilateral paraspinal incisions, release the thoracolumbar fascia, and debride all nonviable muscle. Do not attempt a tight primary closure; instead, apply vacuum-assisted dressings to facilitate edema drainage and plan for delayed secondary closure.
- Optimize postoperative care: Leave subfascial drains in place until output is minimal. Perform serial debridements or irrigations as needed. Delay final wound closure until swelling has subsided and healthy granulation tissue is present. Early mobilization and physical therapy can be initiated once the acute phase has resolved to help restore function.

This algorithm underscores the principle that “time is muscle”—early recognition and intervention are essential to avoid missing a developing compartment syndrome. By acting on the early warning signs (pain out of proportion, unexplained paraspinal firmness, imaging evidence of edema, or rising CK), the care team can prevent a PCS from being mistaken for a routine postoperative issue and thereby avert irreversible muscle damage [5, 6].

### Preventive considerations and risk mitigation

Preventing PCS requires minimizing both intraoperative and postoperative factors that could precipitate excessive pressure in the paraspinal compartment. In the operating room, surgeons should intermittently relax any tubular retractors and limit the duration of extreme muscle retraction to reduce ischemic insult. Although MIS procedures often involve minimal bleeding, it is prudent to insert a small subfascial drain at closure—even if the field appears dry—since occult oozing can otherwise accumulate under the tight fascia. During patient positioning, avoid excessive table flexion or prolonged focal pressure on the back muscles. Intraoperative neuromonitoring of paraspinal muscle oxygenation or pressure (if such technology becomes available in the future) could also help alert the team to developing compartment conditions.

Postoperatively, maintain heightened vigilance in the recovery period. Any patient with disproportionate back pain, abnormally firm paraspinal muscles, new focal sensory deficits, or a rising CK level should prompt immediate re-evaluation, even if the surgery was ostensibly “low-risk” [4, 5]. Particular attention is warranted in young, well-muscled individuals (for example athletes, bodybuilders, military personnel), whose robust paraspinal musculature leaves little extra room within the fascial compartment [4]. Indeed, many reported PCS cases involve young, fit males [4]. These high-risk patients should receive explicit counseling on warning signs and possibly closer postoperative monitoring. While the exact incidence of post-minimally invasive surgery PCS is unknown, a recent review noted only approximately 11 cases reported in the literature to date [4], underscoring how uncommon but serious this complication is. The lessons from this case remind us that “minimally invasive” should not lead to complacency. In summary, we recommend that surgical teams adopt simple precautions (such as intermittent retractor release, prudent use of drains, and limiting operative time), and that postoperative care teams maintain a low threshold for obtaining imaging or surgical consultation when pain seems out of proportion—measures that collectively may prevent a “hidden” compartment syndrome.

### Limitations

Finally, it is important to acknowledge the limitations of this report. As a single case, our observations may not be generalizable to all settings, and our patient’s unique profile (a young, extremely fit male with well-developed paraspinal muscles) may not reflect the typical spine surgery patient. In this case, we did not perform direct compartment pressure measurements at the time of presentation, relying instead on clinical findings, imaging, and intraoperative examination to diagnose PCS. As a result, we were unable to quantify the exact intramuscular pressures or assess their progression over time. Given the rarity of acute paraspinal compartment syndrome, one must exercise caution when extrapolating from this experience. This case highlights the need for heightened awareness, but further reports or case series are needed to better characterize PCS—including its risk factors, time course, and optimal management strategies.

## Conclusion

This case underscores that even minimally invasive pedicle screw fixation can lead to paraspinal compartment syndrome (PCS), a rare but serious complication. Early recognition and differentiation from infection are crucial to prevent treatment delays. In this patient, timely surgical decompression resulted in full recovery, highlighting the importance of prompt intervention. Further studies will be important to elucidate the risk factors and optimal prevention strategies for PCS, given its rarity. This case emphasizes the necessity for heightened awareness of PCS in spine surgery.

## Data Availability

The datasets used and analyzed during the current study are available from these corresponding authors upon reasonable request.

## Abbreviations

MIS-PSF: Minimally invasive percutaneous pedicle screw fixation
PCS: Paraspinal compartment syndrome
WBC: White blood cell
PCT: Procalcitonin
ESR: Erythrocyte sedimentation rate
CRP: C-reactive protein
CK: Creatine kinase
MRI: Magnetic resonance imaging
H&E: Hematoxylin and eosin
